# Influenza and influenza-like syndromes: the subjects’ beliefs, the attitude to prevention and treatment, and the impact in Italian general population

**DOI:** 10.1186/s40248-018-0119-6

**Published:** 2018-03-01

**Authors:** Roberto W. Dal Negro, Alessandro Zanasi, Paola Turco, Massimiliano Povero

**Affiliations:** 1 National Centre for Respiratory Pharmacoeconomics & Pharmacoepidemiology, CESFAR, Verona, Italy; 2 Italian Association for Cough Study (AIST), Bologna, Italy; 3 Research & Clinical Governance, Verona, Italy; 4 AdRes Health Economics and Outcome Research, Turin, Italy

**Keywords:** Influenza, Influenza-like syndromes, Prescribing attitude, Subjects’ beliefs, Survey in general population

## Abstract

**Background:**

Influenza and influenza-like syndromes (I-LSs) are infectious diseases occurring on a seasonal basis which can lead to upper (URTI) and lower respiratory tract illness (LRTI) of different severity. The approach to these disorders is unfortunately not uniform. Aim of the study was to investigate real-life people beliefs, the attitude to their prevention and treatment, and their impact in general population.

**Methods:**

A cross-sectional survey via Computer Assisted Telephone Interview (CATI) was carried out using a specific questionnaire investigating influenza episode rates, subjects behavior in case of influenza and I-LSs, and prescribed therapy.

**Results:**

1,202 subjects completed the questionnaire: median age was 46, 49% male, 20% active smokers. 57% of respondents experienced at least one episode of influenza or I-LS in the previous 12 months; episodes were usually home-managed, shorter than 2 weeks and more frequent in fall and winter (73% of the total). GP resulted the first health-care option (56%); almost 3% of respondents referred to the emergency room, and hospitalization occurred in 1%. Mucolytics resulted the most prescribed drugs (55%) followed by antibiotics and aerosol therapy (37–38%). Even if more than 70% of subjects considered vaccination essential, only 14% received influenza vaccination yearly and almost 60% had never received vaccination. Approximately 36% of respondents regarded homeopathy (namely Oscillococcinum) as an helpful alternative because of perceived as safer.

**Conclusions:**

Seasonal prevalence of I-LSs and influenza partially overlap. As virus identification is not a common procedure in daily practice, only a clinical discrimination is possible. Antibiotic prescription is still too high and largely inappropriate. Influenza vaccination is strongly encouraged, but different strategies are also used. Other approaches are receiving increasing attention in general population, and subjects’ willingness to spend out-of-pocket for effective remedies is also increasing. The discrepancy between subjects’ beliefs and health care actions likely reflects the insufficiency of institutional preventive strategies. In general, the approach to influenza and I-LSs appear variable and highly dependent of subjects’ and their GPs’ cultural beliefs.

## Background

Influenza and influenza-like syndromes (I-LSs) are infectious diseases caused by influenza and other respiratory viruses, and are important causes of upper (URTI) and lower respiratory tract illness (LRTI) [1–7]. Signs of influenza and I-LSs are frequently similar, common symptoms including headache, fever, cough, sore throat, aching muscles and joints, and generally feeling awful [1, 2, 6, 7]. Usually, I-LSs are more severe than a simple cold even in the presence of a mild infection, and represent a frequent cause of general practitioner (GP) consultation by outpatients [8, 9]. Nevertheless, there is a wide range of influenza and I-LSs severity, from the claim of minor symptoms up to the occurrence of severe pneumonia, or encephalitis, or a general whole-body infection: even if infrequent, any of these can be life threatening [1, 2, 6].

The most common respiratory manifestations of influenza (with the epidemic peak usually at the beginning of February) and I-LSs (more widely distributed over the twelve months) are pharyngitis, laryngitis, tracheitis, bronchitis, and pneumonia primarily due to the viral involvement of airway structures [1, 2]**,** but also due to bacterial infections on top of the original viral infection [10]. These events are usually managed at home, but sometimes they require hospitalization and can be life-threatening, particularly in pediatric patients, in the elderly, and in patients already suffering from relevant chronic comorbidities [1, 11–17].

Aim of the study was to investigate and assess real-life beliefs, the attitude to prevention and treatment, and the impact of influenza and I-LSs in the Italian general population.

## Methods

The survey was planned and carried out by CESFAR (National Centre for Respiratory Pharmacoeconomics and Pharmacoepidemiology); AIST (Italian Association for Cough Study), and AdRes (Health Economics and Outcome Research).

The design of the survey was approved by the CESFAR Ethical Committee on October 26^th^, 2016, The survey was conducted between March 5^th^- 21^st^, 2017.

A cross-sectional telephone survey was carried out by means of a specific, validated questionnaire (see Appendix 1) on a representative sample of Italian general population. According to the consolidated validation procedures, the original version of the questionnaire was previously submitted to a sample of 25 individuals (the usual sample size for a pilot test) of different educational levels and randomly chosen in order to check the comprehension of the questions included in the questionnaire. Items where linguistic errors and/or misunderstandings had occurred, were reworded up to their full comprehension.

All interviews were carried out according to the Computer Assisted Telephone Interview (CATI) methodology [18] by expert, professional interviewers. The interviewer was provided with one “work station” consisting of a personal computer connected to a central processing unit. The central unit was also equipped with a specific software for the random choice of individuals (such as, the telephone numbers) to contact. The sampling strategy adopted in the present survey was the random selection of an adequate number of subjects. All interviews were always preceded by a short explanation of the aim of the survey, and had a mean duration of five minutes.

A minimum number of 1,200 respondents was previously calculated in order to collect a representative sample of Italian population in terms of age; gender, job, smoking habit, and national geographical distribution (3% mean effect size; 5% significance level, and 80% statistical power).

Only interviews obtained after the respondent’s informed consent to the interview itself, together to the possible use of information for scientific purposes were considered.

### The questionnaire

A questionnaire was the instrument chosen for collecting information about the subjects’ beliefs, their prevention and therapeutic actions against influenza and/or I-LSs, and the impact of these disorders (Appendix 1). The questionnaire consisted in 21 closed, and five open questions.

The domains of the questionnaire were:

- Demographic characteristics of respondents (age, gender, job, geographical distribution);
- Presence of respiratory comorbidities;
- Convincement on influenza or I-LSs;
- Frequency of influenza or I-LSs episodes in the last 12 months, and their duration;
- Attitude to vaccination and other prevention remedies;
- Therapeutic approach to influenza or I-LSs;
- Severity of episodes (hospital admissions, emergency room accesses);
- Willingness to pay for effective remedies.

### Statistical analysis

Dichotomous and categorical data were reported as absolute (N) and relative (%) frequencies, continuous variables were reported as mean and standard deviation (SD) if normally distributed, otherwise as median and interquantile range (IQR). Normality was tested using Shapiro-Wilk test. To infer quantitative information from categorical answers, the central value of each category was considered (e.g. for answer “less than 7 days” the central value is (1 + 6)/2 = 3.5 days) and weighted mean according to recorded frequencies was calculated; lowest and highest limits of each category were used to estimate a plausible range of variability.

Differences were evaluated using the chi-square test; when samples were small and the assumptions for the chi-square were violated, the Fisher’s exact test could be used. All analyses were performed using computer software R 3.1.2 [19].

## Results

A total of 1,202 respondents completed the interview, and their corresponding questionnaires were analyzed. The overall telephone contacts were 3.978, and the corresponding redemption rate (such as, the proportion of calls properly completed and reliable for the investigation) was 30.2% (1 valid questionnaire every 3.3).

**Fig. 1:**
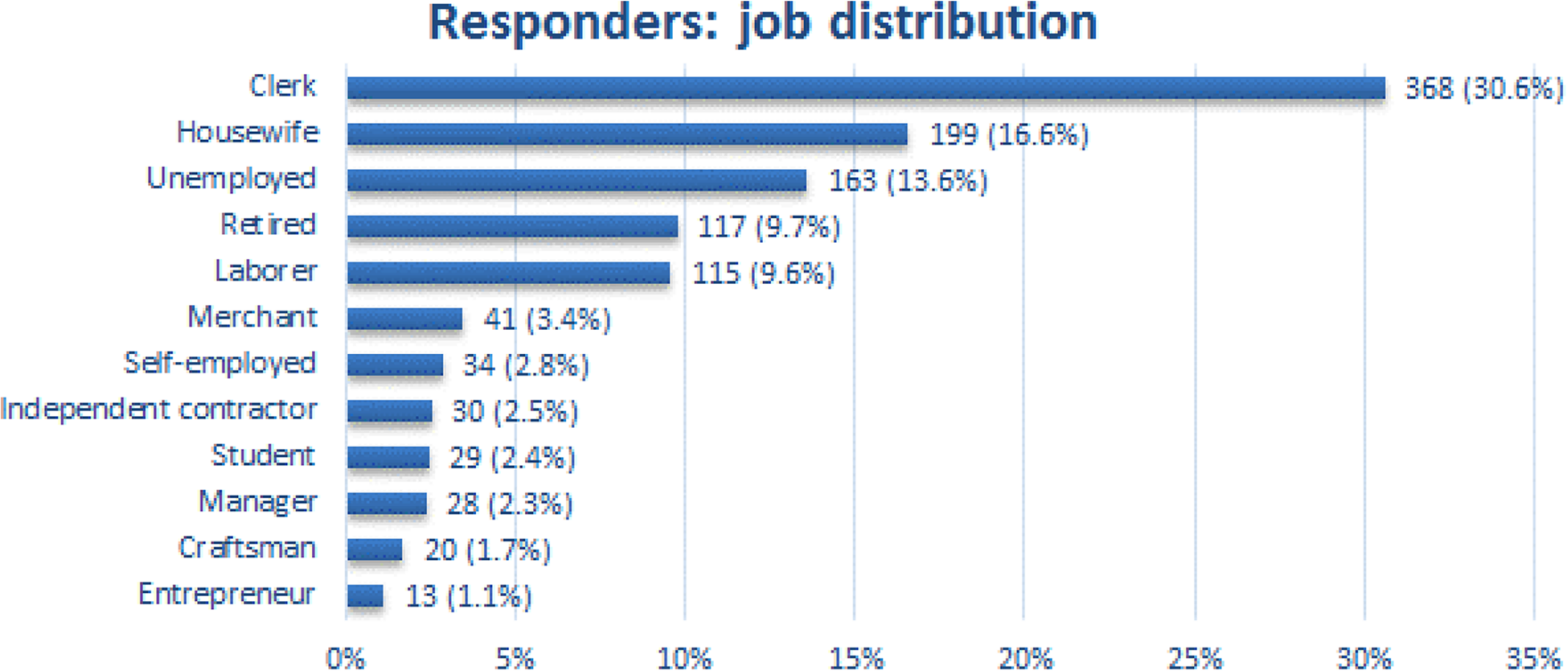
Job distribution of the respondents

Demographic and social characteristics of the sample are reported in Table 1. Responders were in their mid-decade of life, females were slightly prevailing, the geographical distribution was fairly uniform, and the prevalence of active smokers was corresponding to national data. Furthermore, subjects who declared to suffer from a chronic respiratory illness were 6.5%, such as a figure absolutely corresponding to the most recent national prevalence of this kind of respiratory disorders [17]. Concerning the analytic distribution of subjects’ job, clerks (30.6%), housewives (16.6%), unemployed (13.6%), retired persons (9.7%), and laborers (9.6%) were the most frequent respondents (Fig. 1).

**Table 1:** Demographic characteristics of analyzed sample

Baseline characteristics	*N* = 1,202
Age ─ median (IQR)	46 (34─60)
Male ─ N (%)	586 (48.8%)
Chronic respiratory illnesses ─ N (%)	78 (6.5%)
Smoker ─ N (%)	
Active	243 (20.3%)
Ex-smoker	807 (67.3%)
Never	150 (12.5%)
Geographic area ─ N (%)	
North-West	299 (24.9%)
North-Est	304 (25.3%)
Central	262 (21.8%)
South and Islands	337 (28.0%)

*IQR* interquartile range

More than half of respondents (57.2%) complained at least one influenza or I-LS episode over the last 12 months (mean 1.81, range 1.55─2.06); furthermore, duration of each episode was usually lower than one week (mean duration 5.27 days, range 2.41─8.12). 55.7% of respondents, consulted their GP first, and almost 3% referred to the emergency room; hospitalization was required for less than 1% of respondents (Table 2).

**Table 2:** Main characteristics of influenza or I-LSs episodes registered in the questionnaire

	Total *N* = 1,202
Respondents with influenza or I-LSs episodes	688 (57.2%)
Episodes per year ─ mean (range)	1.81 (1.55; 2.06)
1 episode ─ N (%)	356 (52.4%)
2–3 episodes ─ N (%)	298 (43.9%)
> 3 episodes^a^ ─ N (%)	25 (3.7%)
Duration ─ mean (range)	5.27 (2.41; 8.12)
< 7 days ─ N (%)	526 (76.5%)
7–15 days ─ N (%)	162 (23.5%)
Subjects requiring GP ─ N (%)	383 (55.7%)
Subjects requiring ER ─ N (%)	19 (2.8%)
Hospitalizations ─ N (%)	6 (0.9%)
< 7 days ─ N (%)	3 (50.0%)
16–30 days ─ N (%)	3 (50.0%)

*I-LSs* influenza-like syndromes, *GR* general practitioner, *ER* emergency room

^a^A maximum number of 6 episodes per year was assumed; this limit was estimated fitting a Poisson distribution on the frequencies recorded in the questionnaires

Results of subgroup analyses did not show a statistically significant association between these outcomes and administration of annual vaccination or smoking status (Table 3). Annual vaccination seems to reduce infectious events (1.72 vs 1.83 for subjects never vaccinated) but the reduction did not reach statistical significance (*p* = 0.63). According to smoking status, hospitalization rate is lower in smokers with respect to no or ex-smokers (*p* < 0.01).

**Table 3:** Main characteristics of influenza or I-LSs episodes registered in the questionnaire according to vaccination and smoking status

	Did you receive annual vaccination?				Smoking status			
	Never *N* = 685 (57%)	Sometimes *N* = 335 (30%)	Yes *N* = 170 (14%)	*p*	Smokers *N* = 243 (20%)	Never smoke *N* = 807 (67%)	Ex-smokers *N* = 150 (13%)	*p*
Respondents with influenza or I-LSs episodes	393 (57.4%)	185 (55.2%)	102 (60.0%)	0.58	136 (56.0%)	470 (58.2%)	80 (53.3%)	0.49
Episodes per year ─ mean (range)	1.83 (1.57; 2.10)	1.77 (1.52; 2.02)	1.72 (1.49; 1.95)	0.63	1.78 (1.53; 2.03)	1.82 (1.56; 2.08)	1.73 (1.49; 1.96)	0.94
1 episode ─ N (%)	198 (50.8%)	97 (53.0%)	58 (58.6%)		73 (54.1%)	239 (51.6%)	44 (55.7%)	
2–3 episodes ─ N (%)	177 (45.4%)	81 (44.3%)	37 (37.4%)		57 (42.2%)	206 (44.5%)	33 (41.8%)	
> 3 episodes^a^ ─ N (%)	15 (3.8%)	5 (2.7%)	4 (4.0%)		5 (3.7%)	18 (3.9%)	2 (2.5%)	
Duration ─ mean (range)	5.16 (2.33; 7.99)	5.20 (2.36; 8.04)	5.71 (2.76; 8.65)	0.29	5.10 (2.28; 7.92)	5.27 (2.42; 8.13)	5.56 (2.65; 8.48)	0.59
< 7 days ─ N (%)	306 (77.9%)	143 (77.3%)	72 (70.6%)		107 (78.7%)	359 (76.4%)	58 (72.5%)	
7–15 days ─ N (%)	87 (22.1%)	42 (22.7%)	30 (29.4%)		29 (24.4%)	111 (23.6%)	22 (27.5%)	
Subjects requiring GP ─ N (%)	212 (53.9%)	107 (57.8%)	60 (58.8%)	0.54	74 (54.4%)	269 (57.2%)	39 (48.8%)	0.35
Subjects requiring ER ─ N (%)	8 (2.0%)	7 (3.8%)	4 (3.9%)	0.37	4 (5.7%)	12 (4.5%)	3 (7.7%)	0.83
Hospitalizations ─ N (%)	1 (0.3%)	1 (0.5%)	4 (3.9%)	0.08	0 (0.0%)	3 (0.6%)	3 (3.8%)	0.01
< 7 days ─ N (%)	0 (0%)	0 (0%)	3 (75.0%)		0 (0.0%)	1 (33.3%)	2 (66.7%)	
16–30 days ─ N (%)	1 (100.0%)	1 (100.0%)	1 (25.0%)		0 (0.0%)	2 (66.7%)	1 (33.3%)	

*I-LSs* influenza-like syndromes, *GR* general practitioner, *ER* emergency room

^a^A maximum number of 6 events per year was assumed; this limit was estimated fitting a Poisson distribution on the frequencies recorded in the questionnaires

**Fig. 2:**
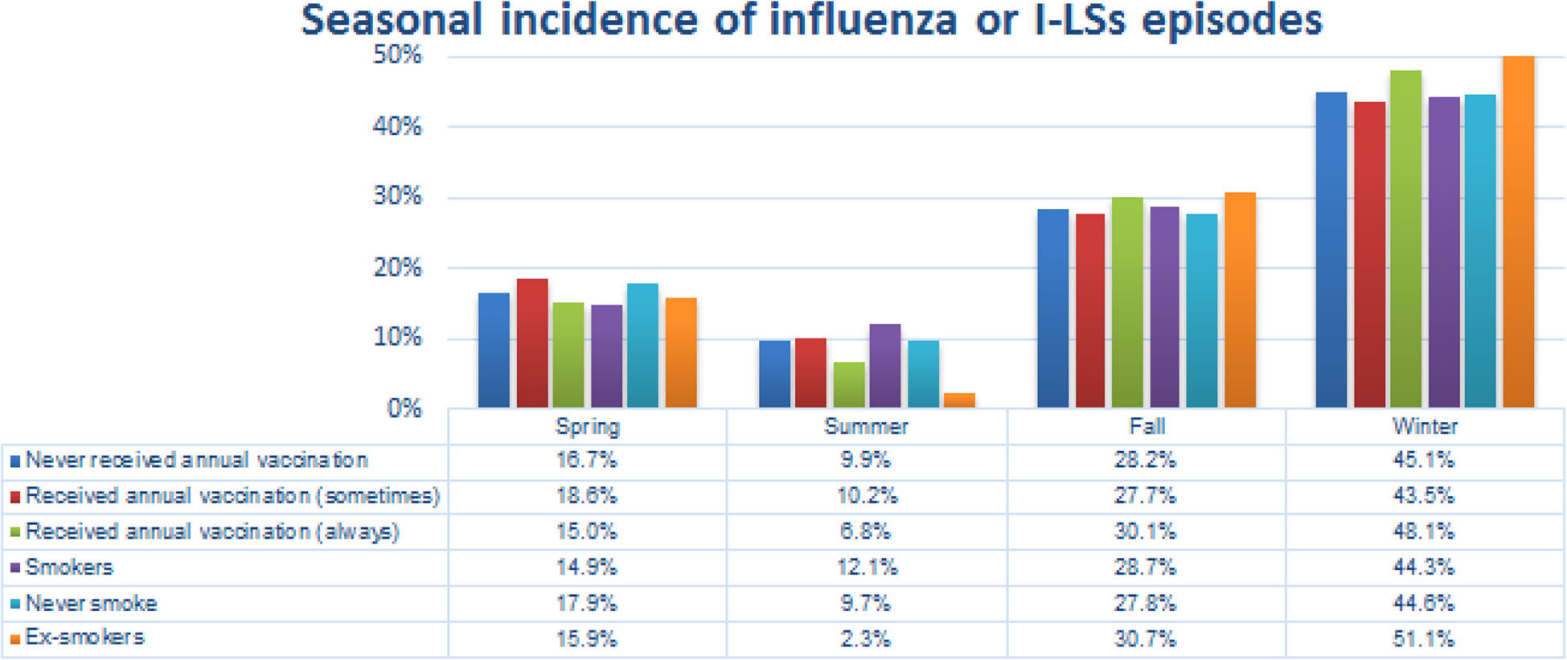
Incidence of influenza or I-LSs episodes in the different seasons according to vaccination and smoking status

**Fig. 3:**
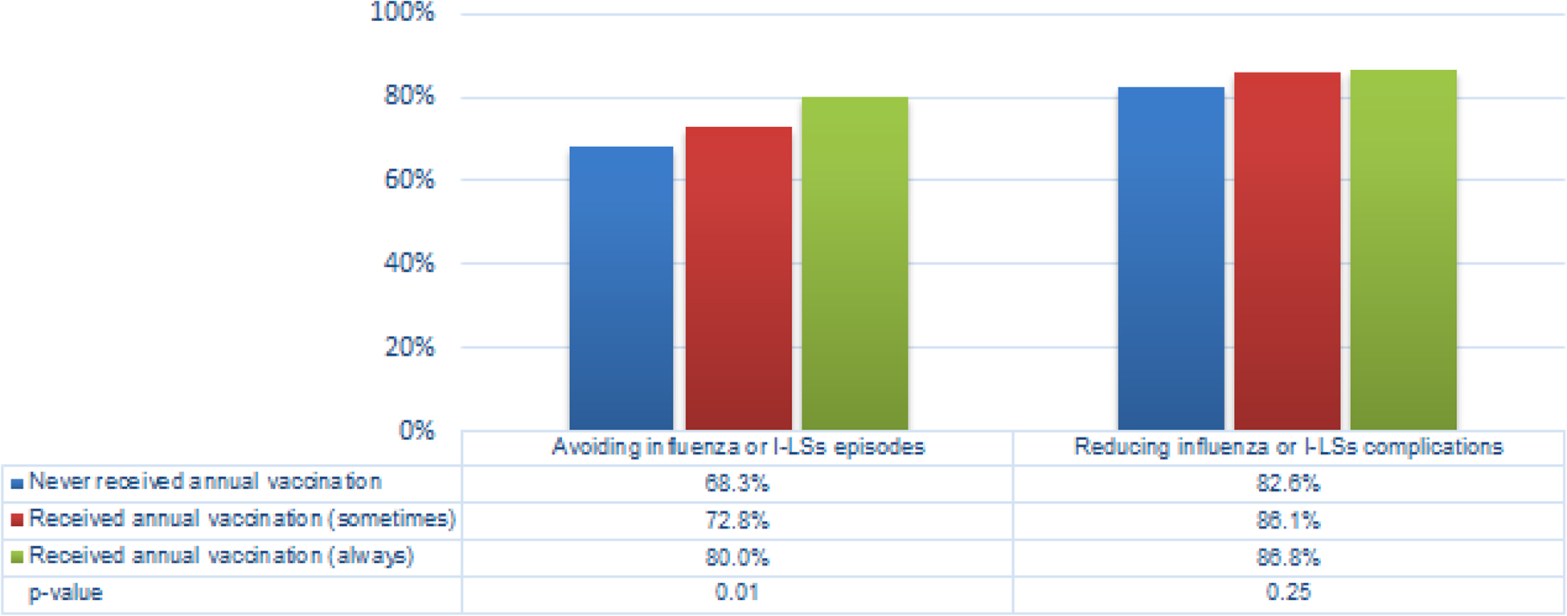
Subjects'beliefs on vaccination

As expected, most of the episodes occurred during fall (28.3%) and winter (45.1%); seasonal incidence did not depend on vaccination status (*p* = 0.85) while it varied according to smoking status (*p* = 0.01) with a slightly high incidence of episodes in winter for ex-smokers (> 50%, p = 0.01) (Figs. 2 and 3).

The overall GPs’ therapeutic approach and the classes of drugs prescribed are reported in Table 4 for subjects experienced at least one influenza or I-LS episode and for subjects without any episodes. It seems that there was no difference in GP prescriptions for subjects with or without influenza or I-LSs, except for oral corticosteroids (4.3% in case of influenza vs 1.8%, *p* = 0.03).

**Table 4:** Drugs prescribed by general practitioners

Respondents receiving a prescription of	With influenza or I-LSs episodes	Without influenza or I-LSs episodes	*p*
Antibiotics ─ N(%)	253 (37.6%)	172 (34.4%)	0.90
Oral corticosteroids ─ N(%)	29 (4.3%)	9 (1.8%)	0.03
Non-steroidal anti-inflammatory drugs ─ N(%)	199 (29.5%)	154 (30.4%)	0.79
Mucolytic and/or cough suppressants ─ N(%)	373 (55.1%)	275 (54.9%)	0.99
Aerosol therapy ─ N(%)	247 (36.5%)	177 (34.8%)	0.57

*I-LSs* influenza-like syndromes

In terms of prevention and prophylaxis, the vast majority of respondents affirmed to regard vaccination as an helpful choice for avoiding influenza (almost 80% for patients receiving annual vaccination, *p* = 0.01) and to reduce possible complications (84.3% with no differences according to vaccination status, *p* = 0.25). A smaller, but not negligible, proportion of respondents were instead convinced that vaccination is ineffective (13.4%), or even dangerous (11.6%).

**Fig. 4:**
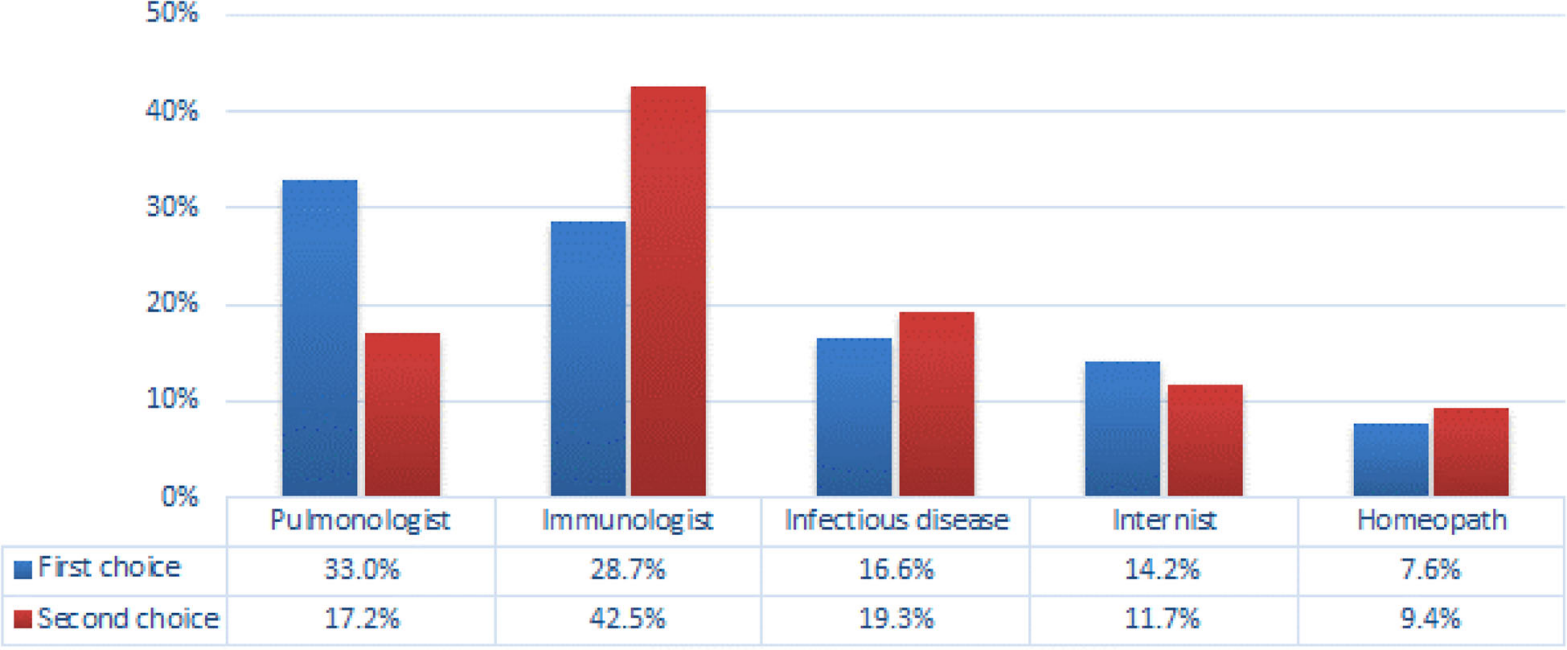
The preferred specialist presumed to be contacted in case of persistence or aggravation of influenza or I-LSs episodes

In case of persistence or aggravation of symptoms, almost one third of the respondents would contact a pulmonologist first. The immunologist resulted the most frequent second choice, followed by the Specialist of Infectious Disease and the Internist, respectively. Also the Homeopath is consulted in these circumstances (7.6% as first and 9.4% as second option) (Fig. 4).

A section of the questionnaire was oriented to assess the subjects’ attitude to homeopathic drugs. Almost 64% of responders never used homeopathic drugs, neither for prevention, nor for treating influenza or I-LSs (Table 5), while 36% of responders declared to use at least occasionally homeopathic drugs. About 30% think that such medications could be helpful in preventing influenza or I-LSs, and seem safer than regular vaccination. However, a substantial uncertainty on this topic is clearly mirrored by the high proportion of subjects who did not provide their opinion (more than 30%).

**Table 5:** Use and attitude towards homeopathic drugs for the prevention and treatment of influenza or I-LSs events

Use and attitude	N (%)		
Never used	759 (63.9%)		
Occasionally/usually/always used	429 (36.1%)		
Do you think that homeopathic drugs are…	Yes	No	Doubtful
helpful in preventing IEs?	370 (30.8%)	429 (35.7%)	103 (33.5%)
Safer than vaccination?	343 (28.5%)	490 (40.8%)	369 (30.7%)

*I-LSs* influenza-like syndromes

As concerning the subjects’ willingness to spend, respondents claimed willing to spend an average of € 13 for an effective influenza or I-LSs prevention (range 8–17 €); in particular, more than 70% of respondents would be willing to spend up to 20 € (Table 6); no differences were detected between respondents that experienced or not influenza or I-ILs episodes in the last 12 month (*p* = 0.22).

**Table 6:** Willingness to pay for effective prevention against influenza or I-LSs episodes

	Total	With influenza or I-LSs episodes	Without influenza or I-LSs episodes	*p*
Nothing ─ N (%)	249 (22.8%)	146 (23.0%)	103 (22.6%)	
< 10 € ─ N (%)	251 (23.0%)	155 (24.4%)	96 (21.1%)	
10–20 € ─ N (%)	346 (34.5%)	219 (34.5%)	157 (34.5%)	
20–30 € ─ N (%)	120 (11.0%)	65 (10.2%)	55 (12.1%)	
> 30 € ─ N (%)	94 (8.6%)	50 (7.9%)	44 (9.7%)	
Mean (range)	€ 12.64 (8.47–16.82)	€ 12.22 (8.10–16.35)	€ 13.23 (8.98–17.47)	0.22

*I-LSs* influenza-like syndromes

## Discussion

Influenza and influenza-like syndromes (I-LSs) are characterized by a wide spectrum of respiratory and non-respiratory manifestations, and can frequently lead to upper (URTI) and lower respiratory tract illness (LRTI) [1–9]. They are very common events and a large proportion of general population is yearly involved, even if at different degrees of severity. Specific vaccination against influenza is strongly encouraged because it represents the very first action, effective for containing the spreading, the clinical impact, and the burden of the disease, in particular in elderly, in pediatric age, and in subjects at risk, or suffering from dangerous comorbidities [1, 16, 20]. Furthermore, early vaccination was recently proved to be effective also in reducing the frequency of I-LSs [21]. However, some specific anti-viral drugs are also available in the aim to prevent influenza and I-LS and/or to manage their symptoms, and some new molecules are still in development [10, 15, 22–25].

Even if several drugs are prescribed for treating influenza and I-LSs in adults and children, only few studies were dedicated to investigate the therapeutic habits usually taken in real life by general population against these infectious events [26].

Data of the present national survey contributed to depict a real-life global framework on this topic, just focusing some aspects concerning the subjects’ attitude to prevention against influenza and I-LSs, their beliefs, their usual behavior, and the prescriptions they receive in these circumstances.

Once again influenza and I-LSs confirmed to represent very common infections as more than half of respondents did suffer from at least one episode over the last 12 months. Unfortunately, the identification of the specific virus involved is not a common procedure in daily clinical practice, and the etiology of these infections can then be mostly presumed only on the basis of their seasonal occurrence and clinical signs claimed by subjects. Actually, while influenza usually prevails in late Fall-beginning Winter and is epidemic, I-LSs (namely, those infections due to other respiratory viruses) have a much more scattered occurrence over the year, and they also prevail not only in fall and winter but also in spring and summer. Data of the present survey confirm this wider distribution, and they pinpoint how much the prevalence of I-LSs can also be relevant during the year, even though partially overlapping with that one of influenza. Furthermore, likely due to the mean age of respondents, the clinical manifestations of these events had in general been reported as not severe, and, as in previous studies [27], only a very small proportion of respondents required emergency visits or hospital admission also in the present survey.

Actually, these events were mostly managed at home by means of symptomatic remedies only, and mucolytics and/or cough suppressants were the drugs most frequently prescribed, merely in the aim to alleviate symptoms. While systemic corticosteroids were not frequently prescribed, the frequency of antibiotic prescription recorded in the present survey resulted high indeed (more than in 35% of cases). This figure confirms the high and inappropriate attitude in antibiotic prescribing in primary care, which was also reported in previous studies carried out in different countries, independently of the subjects’ age [25, 27–30]. To note that also the National Guidelines for the management of influenza-like syndrome in adults and children do not recommend the use of antibiotics in non-complicated conditions, unless the clinical picture is evident for proven bacterial co-infections [31].

When considering the low degree of severity of the infectious events generally reported in the present survey, the corresponding extent of antibiotic prescription can be easily presumed as exceeding and largely inappropriate, if not useless and dangerous, at least in terms of the potential increase of bacterial resistances induced [29, 30]. Unfortunately, if this inappropriate use of antibiotics in these circumstances reflects a not negligible proportion of self-medication [25], in the majority of cases it is related to a consolidated negative attitude in primary care which is difficult to be modified and which is likely due to a non-specific aspect of defensive medicine.

In terms of prevention and prophylaxis, even if the majority of respondents declare to consider vaccination as an useful choice for both avoiding influenza and for reducing possible related complications, nevertheless a small proportion of subjects receive influenza vaccination every year, and the majority of subjects never experienced vaccination at all. This aspect results conflicting with respondents’ claimed beliefs and likely reflects the insufficiency of institutional actions in favor of a specific and penetrating information, or the substantial practical ineffectiveness of vaccination campaign, or the insufficient empowerment and accountability of primary care.

The hypothesis that this discrepancy would be due to organizational and cultural failures seem confirmed by the high number of subjects who adopt preventive actions different from vaccination, and assume oral drugs on regular basis with preventive purposes, mostly against I-LSs [31, 32]. Also the position of a not negligible proportion of respondents whose beliefs are against vaccination (namely regarded as a dangerous or harmful medical procedure) is likely related to insufficient communication and empowerment of general population.

On the other hand, other approaches have been proposed, even if with still debated results. In particular, vitamin supplementation and herbal remedies did not prove any significant results when evaluated in controlled studies vs placebo [33, 34].

It cannot be forgotten that approximately one third of respondents claimed positive beliefs towards the homeopathic approach to I-LSs. In general, it was described that patients managed by GPs certified in homeopathy used less antibiotics and anti-inflammatory drugs for I-LSs than those managed by GPs prescribing conventional medications [26], but this effect was not explained and no hypothesis was provided for supporting this significant difference.

In a Cochrane review of a few years ago, the effect of Oscillococcinum (such as a patented, commercially available homoeopathic drug obtained from wild duck heart and liver, which are regarded to be reservoirs for influenza viruses) was compared to placebo. The review concluded that, though promising, data were not strong enough to support a general recommendation to use this drug in first-line treatment of influenza or I-LSs [35].

However, another review, even though postulating the need of further controlled studies, concluded that the effect of Oscillococcinum is not inferior to conventional treatment in pragmatic equivalence trials carried out in primary care [36]. Furthermore, a recent retrospective investigation showed that Oscillococcinum was able to cause a significant reduction of I-LSs episodes over the study period, and its positive effect in preventing I-LSs was then suggested [37].

Influenza and I-LSs have a great social impact and generate high direct and indirect costs to the institutions. These costs are related either to health interventions for acute conditions and/or to associated infections or complications, both in adults (particularly in elderly and subjects at risk), and children [1, 9, 10, 13, 31].

Independently of the different therapeutic strategy adopted for preventing and containing the effects these illness, many unmet needs still remain [21]. This particular aspect can also be easily perceived by the evidence that more than 70% of respondents claimed willing to spend up to 20 € out-of-pocket for an effective influenza or I-LSs preventive intervention. This point is worthy to be analyzed more in depth. In particular, it would be interesting to estimate the actual expense that families have to bear for treating a case of influenza or ILS, and compare it with their willingness to pay for prevention.

## Conclusions and limitations of the study

Influenza and influenza-like syndromes (I-LSs) are very common events within general population with respiratory and non-respiratory manifestations of different clinical severity, and substantial economic burden for both people and health institutions.

Several interventions are adopted with different results, but people’s convincement and approach are quite uneven. The vaccination rate still is insufficiently used in general population, and, unfortunately, a not negligible proportion of subjects is still convinced that it is useless or dangerous. Obviously, more effective strategies are needed, as well as more clear and deep cultural messages which should reach all levels of general population.

As for the pharmacological options mostly used in real life, antibiotics still remain over-prescribed, without any supporting evidence in the vast majority of cases. The aerosol route for drug administration is frequently used. Moreover, a not negligible proportion of subjects affirm their interest/curiosity versus homeopathic drugs.

The present survey has some limitations. Influenza and I-LSs diagnosis were only presumed by subjects claims, and corresponding viruses were not identified, as occurring in the great majority of cases in real life. Even if likely over prescribed on the basis of the general low degree of severity of events reported, antibiotics were not identified in terms of molecules, doses, and duration of treatment. The appropriateness of their use will be further assessed in more specific studies, already started.

However, independently of the different options presently available on the market, the general approach to, and the management of influenza and I-LSs appear to be variable and highly dependent of subjects’ and their GPs’ cultural convincement.

## Abbreviations

CATI: Computer Assisted Telephone Interview
GP: General practitioner
I-LSs: Influenza-like syndromes
LRTI: Lower respiratory tract illness
SD: Standard deviation
URTI: Upper respiratory tract illness

## References

[1] European Centre for Disease Prevention and Control, Questions and answers on seasonal influenza ─ http://ecdc.europa.eu/en/healthtopics/seasonal_influenza/Pages/QA_seasonal_influenza.aspx

[2] Liu WK, Liu Q, Chen DH, Liang HX, Huang WB, Qin S (2013). Epidemiology and clinical presentation of the four human parainfluenza virus types. BMC Infect Dis.

[3] Saliba W, Fediai A, Edelstein H, Markel A, Raz R (2013). Trends in burden of infectious disease hospitalizations among the elderly in the last decade. Eur J Intern Med.

[4] Gautret P, Gray GC, Charrel RN, Odezulu NG, Al-Tawfig JA, Memish ZA ZA (2014). Emerging viral respiratory tract infections—environmental risk factors and transmission. Lancet Infect Dis.

[5] Bellido-Blasco JB, Pardo-Serrano F, Ballester-Rodriguez I, Arnedo-Pena A, Tirado-Balaguer MD, Romeo-Garcia MA (2015). An estimate of the incidence of influenza-like illness during the influenza pandemic of 2009. Arch Bronchopneumol.

[6] Simon E, Long B, Koyfman A (2017). Clinical mimics; an emergency medicine-focused review of influenza mimics. J Emerg Med.

[7] Ljiaz M, Jaffar Khan M, Khan J, Usama U (2017). Association of clinical characteristics of patients presenting with influenza like illness or severe acute respiratory illness with development of acute respiratory distress syndrome. Monaldi Arch Chest Dis.

[8] Fu Y, Pan L, Sun Q, Zhu W, Zhu L, Ye C (2015). The clinical and etiological characteristics of influenza-like illness (ILI) in outpatients in shanghai, China, 2011-2013. PLoS One.

[9] Giannattasio A, Lo Vecchio A, Napolitano C, Di Florio L, Guarino A (2014). A prospectic study on ambulatory care provided by primary care pediatricians during influenza season. Ital J Pediatr.

[10] No Authors listed. Antiviral drugs in influenza: an adjunct to vaccination in some situations. Prescrire Int. 2006;15:21–30. 16548114

[11] Marzano A, Marengo A, Ruggiero T, Allice T, Sanna C, Alessandria C (2013). Clinical impact of a/H1/N1/09 influenza in patients with cirrhosis: experience from a nosocomial cluster of infection. J Med Virol.

[12] Mikulska M, Del Bono V, Gandolfo N, Dini S, Dominietto A, Di Grazia C (2014). Epidemiology of viral respiratory tract infections in an outpatient hematology facility. Ann Hematol.

[13] Albarran-Sanchez A, Ramirez-Renteria C, Huerta-Montiel F, Martinez-Jeronimo A, Herrera-Landero A, Garcia-Alvarez JL (2016). Clinical features of patients with influenza-like illness who went to a third level center in the winter of 2013-2014. Rev Med Inst Mex Seguro Soc.

[14] Bulgakova VA, Poromov AA, Grekova AI, Pshenichnaya NY, Selkova EP, Lvov NI (2017). Pharmacoepidemiological study of the course of influenza and other acute respiratory viral infections in risk groups. Ter Arkh.

[15] Lehners N, Tabatabai J, Prifert C, Wedde M, Puthenparambil J, Weissbrich B (2016). Long-term shedding of influenza virus, parainfluenza virus, respiratory syncytial virus and nosocomial epidemiology in patients with hematological disorders. PLoS One.

[16] Ministry of Health, Influenza Portal ─ http://www.salute.gov.it/portale/influenza/homeInfluenza.jsp

[17] Ferrante G, Baldissera S, Campostrini S. On behalf of the PASSI coordinating study group. Epidemiology of chronic respiratory diseases and associated factors in the adult Italian population. Eur J Pub Health. 2017; 10.1093/eurpub/cks109. [Epub ahead of print] 10.1093/eurpub/ckx109 29016794

[18] Core Team R (2013). R: a language and environment for statistical computing.

[19] Kraut R, Brynin M, Kiesler S (2006). Computers, phones, and the internet: domesticating information technology.

[20] Lee A, Chuh A (2010). Facing the threat of influenza pandemic – roles and implications to general practitioners. BMC Public Health.

[21] Saadeh-Navarro E, Garza-Gonzalez E, Salazar-Montalvo RG, Rodriguez-Lopez JM, Mendoza-Flores L, Camacho-Ortiz A (2016). Association between early vaccination and the reduction of influenza-like syndromes in health care providers. Am J Infect Control.

[22] Hayden FG (2013). Advances in antivirals for non-influenza respiratory virus infections. Influenza Other Respir Viruses.

[23] Mok CK, Kang SS, Chan RW, Yue PY, Mak NK, Poon LL (2014). Anti-inflammatory and antiviral effects of indirubin derivates in influenza a (H5N1) virus infected primary human peripheral blood-derived macrophages and alveolar epithelium cells. Antivir Res.

[24] Hermann T (2016). Small molecules targeting viral RNA. Wiley Interdiscip Rev RNA.

[25] Lee-Trilling VT, Megger DA, Katschinski B, Landsberg CD, Ruckborn MU, Tao S (2016). Broad and potent antiviral activity of the NAE inhibitor MLN4924. Sci Rep.

[26] Eckel N, Sarganas G, Wolf IK, Knopf H (2014). Pharmacoepidemiology of common colds and upper respiratory tract infections in children and adolescent in Germany. BMC Pharmacol Toxicol.

[27] Grimaldi-Bensoua L, Begaud B, Rossignol M, Avouac B, Lert F, Rouillon F (2014). Management of upper respiratory tract infections by different medical practices, including homeopathy, and consumption of antibiotics in primary care: the EP13 cohort study in France 2007-2008. PLoS One.

[28] Rezal RS, Hassali MA, Alrasheedy AA, Saleem F, Yusof FA, Kamal M (2015). Prescribing pattern for upper respiratory tract infections: a prescription-review of primary care practice in Kedah, Malaysia. Expert Rev Anti-Infect Ther.

[29] Run Siguroarddottir N, Nielsen AB, Munck A, Bierrum L. Appropriateness of antibiotic prescribing for upper respiratory tract infections in general practice: comparison between Denmark and Iceland. Scand J Prim Health Care. 2105;33:269–74. 10.3109/02813432.2015.1114349 PMC4750736 26683287

[30] Tyrstrup M, van der Velden A, Engstrom S, Goderis G, Molstad S, Verheij T (2017). Antibiotic prescribing in relation to diagnoses and consultation rates in Belgium, the Netherlands and Sweden: use of European quality indicators. Scand J Prim Health Care.

[31] Morciano C, Vitale A, De Masi S, Sagliocca L, Sampaolo L, Lacorte E (2009). Italian evidence-based guidelines for the management of influenza-like syndrome in adults and children. Ann Ist Super Sanità.

[32] Braido F, Tarantini F, Ghiglione V, Melioli G, Canonica GW. Bacterial lysate in the prevention of acute exacerbation of COPD and in respiratory recurrent infections. Int J COPD. 2007;2:335–45. PMC2695191 18229572

[33] Rees JR, Hendricks K, Barry EL, Peacock JL, Mott LA, Sandler RS (2013). Vitamin D3 supplementation and upper respiratory tract infections in a randomized, controlled trial. Clin Infect Dis.

[34] Wong LY, Leung PC, Pang SY, Cheng KF, Wong CK, Lam WK (2013). A herbal formula for prevention of influenza-like syndrome; a double-blind randomized clinical trial. Clin J Integr Med.

[35] Vickers AJ, Smith C (2006). Homoeopathic Oscillococcinum for preventing and treating influenza and influenza-like syndromes. Cochrane Database Syst Rev.

[36] Bellavite P, Marzotto M, Chirumbolo S, Conforti A (2011). Advances in homeopathy and immunology: a review of clinical research. Front Biosci.

[37] Beghi GM, Morselli-Labate AM (2016). Does homeopathic medicine have a preventive effect on respiratory tract infections? A real life observational study. Multidiscip Respir Med.

